# Could Hepatitis B Reactivation be Overlooked in Patients with Resolved Hepatitis B Virus Infection and Receiving Immunosuppressive Treatment?

**DOI:** 10.5152/tjg.2024.23534

**Published:** 2024-04-01

**Authors:** Şencan Acar, Ahmet Tarık Eminler

**Affiliations:** 1 Department of Gastroenterology, Amasya University Sabuncuoğlu Şerefeddin Training and Research Hospital, Amasya, Turkey; 2 Department of Gastroenterology, Sakarya University Faculty of Medicine, Sakarya, Turkey

Dear Editor,

I read with great interest the study by Han et al^1^ entitled “Risk of hepatitis B reactivation in patients with resolved infection on therapy with corticosteroids and conventional synthesis immunosuppressants for kidney disease: a single-center analysis of 258 patients.”

The study group included 258 cases with resolved hepatitis B virus (HBV) infection, all of whom received steroids, with 74.4% also receiving conventional synthesis immunosuppressants. At baseline, all of the patients had a negative HBV DNA, and 73% tested positive for anti-HBs (ranging from 10.4-970, with a mean of 144.7 mIU/mL). Although liver function tests were monitored monthly in all patients, HBV DNA was monitored every 1-3 months in less than half (41.1%) of them. The authors observed no HBV reactivation (HBVr) during the study period. However, 14 patients experienced seroconversion from a positive anti-HB status to a negative status. Finally, the authors concluded that universal prophylaxis may not be justified or cost-effective in patients with a resolved infection.

In individuals exposed to HBV, the risk of HBVr during immunosuppressive treatment (IST) depends on host-related factors, virological factors, and the IST protocol. The IST protocol used in cases involving B-cell-depleting agents and stem cell and solid organ transplantation increases the risk of HBVr. Corticosteroids are also considered high-risk agents after these 2 IST protocols. It is well known that HBVr has 3 phases; phase 1 involves increased HBV replication, phase 2 involves hepatic damage, and phase 3 involves a recovery period. Alanin aminotranspherase (ALT) levels may not increase or may increase minimally in phase 1. Therefore, without examining the hepatitis B surface agent (HBsAg) or HBV DNA, it cannot be concluded that there is no reactivation in these patients. Moreover, the presence of a positive baseline anti-HBS status with high titers might have contributed to these HBVr rates, although this is not the case for all patients in clinical practice. Negative anti-HB titers turning positive in patients with no reactivation need to be explained.^2^

A recent study, conducted on 221 HBsAg-negative and anti-HBc positive patients using biologic agents associated with immunomodulatory agents in 152 patients and corticosteroids in 84 patients, also reported HBVr in 2 patients.^3^ In patients with HBsAg (−) and anti-HBc IgG (+), using a moderate dose (10-20 mg/day) or a high dose (>20 mg/day) of prednisone for 4 weeks is considered to pose a medium risk for HBVr, with the HBVr risk ratio ranging from 1% to 10%. Prophylaxis, or preemptive treatment, is recommended for these patients.^4^ Moreover, if there is a delay in the diagnosis of HBV, severe or fulminant hepatitis may develop. A study reported a mortality rate of 17.5% even when a third-generation antiviral treatment was started after the HBV flare had developed.^5^

Another issue worth mentioning is the change in HBV markers after IST. Interestingly, the authors reported seroconversion from a negative anti-HB status to a positive one in 9 patients. 

The fact that negative anti-HB titers turned positive in the patient group who had no reactivation needs to be explained.

In conclusion, there is a considerable risk of HBVr in patients with resolved HBV infection after receiving steroid treatment. Although universal prophylaxis may not be justified, all patients receiving IST should be screened and monitored for reactivation for a minimum of 6 months after discontinuation of IST. 
